# *Sorghum bicolor* x *S. halepense* interspecific hybridization is influenced by the frequency of 2n gametes in *S. bicolor*

**DOI:** 10.1038/s41598-019-53193-3

**Published:** 2019-11-29

**Authors:** George L. Hodnett, Sara Ohadi, N. Ace Pugh, Muthukumar V. Bagavathiannan, William L. Rooney

**Affiliations:** 1 Department of Soil and Crop Sciences, Texas A&M University, College Station, TX 77843-2474 USA; 2 Present Address: Department of Plant Sciences, University of California, Davis, CA 95616 USA; 3 Present Address: School of Plant Sciences, University of Arizona, Tucson, AZ 85721 USA

**Keywords:** Cytological techniques, Evolutionary genetics

## Abstract

Tetraploid johnsongrass [*Sorghum halepense* (L.) Pers.] is a sexually-compatible weedy relative of diploid sorghum [*Sorghum bicolor* (L.) Moench]. To determine the extent of interspecific hybridization between male sterile grain sorghum and johnsongrass and the ploidy of their progeny, cytoplasmic (CMS), genetic (GMS) and chemically induced male sterile lines of Tx623 and Tx631 were pollinated with johnsongrass pollen. At maturity 1% and 0.07% of the developing seeds of Tx623 and Tx631 respectively were recovered. Ninety-one percent of recovered hybrids were tetraploid and two percent were triploid, the tetraploids resulting from 2*n* gametes present in the sorghum female parent. Their formation appears to be genotype dependent as more tetraploids were recovered from Tx623 than Tx631. Because a tetraploid sorghum x johnsongrass hybrid has a balanced genome, they are male and female fertile providing opportunities for gene flow between the two species. Given the differences in 2n gamete formation among Tx623 and Tx631, seed parent selection may be one way of reducing the likelihood of gene flow. These studies were conducted in controlled and optimum conditions; the actual outcrossing rate in natural conditions is expected to be much lower. More studies are needed to assess the rates of hybridization, fitness, and fertility of the progeny under field conditions.

## Introduction

Unreduced gametes (2n gametes) contain the same number of chromosomes as the somatic cells, are widely reported among many plant species and are considered a primary mechanism of polyploidization^1,2^. The occurrence of 2n gametes has also been of significant value in crop improvement. For example, modern sugarcane varieties descend from *Saccharum officinarum* L. x *Saccharum spontaneum* L. hybrids which required 2n gamete transmission from *S. officinarum*. Modern sugarcane varieties possess high sucrose and low fiber content inherited from *S. officinarum* and resistance to the diseases and insect pests that threatened the industry in the early 1900s from *S. spontaneum*^3^. Breeders of potato (*Solanum tuberosum* L.) use 2n gametes to enable the creation of interspecific, interploidy hybrids of *Solanum* that have been used extensively to develop high yielding disease and pest resistant cultivars of the tetraploid cultivated species^4^.

While 2n gametes are beneficial for crop improvement, they can in certain situations facilitate outcrossing between cultivated species and their weedy relatives. Johnsongrass [*Sorghum halepense* (L.) Pers.], a sexually-compatible weedy relative of cultivated sorghum [*Sorghum bicolor* (L.) Moench] commonly inhabits sorghum producing regions of the United States and throughout the world^5^. Cytogenetically, sorghum is a diploid of 2n = 2x = 20 where 2n is the somatic chromosome number having two complete sets (2x) of chromosomes and a chromosome number of 20. Johnsongrass is a tetraploid (2n = 4x = 40). In both species the number of chromosomes in each set is ten. The term “n” represents chromosomes in a haploid cell examples being sperm and egg cells whose chromosomes have been reduced by half during meiosis. In the case of a haploid cell of diploid sorghum n = x = 10 while a haploid cell of tetraploid johnsongrass is n = 2x = 20. The ploidy difference between the two species reduces but does not eliminate interspecific hybridization. In crosses involving *S. halepense* and *S. bicolor*, both triploid and tetraploid progenies have been reported^6–9^. Triploid hybrids were male sterile with very low levels of female fertility, but can survive winters due to vigorous rhizomes^8^. Tetraploids are assumed to develop from the union of an unreduced (2n = 2x = 20) sorghum gamete and a reduced (n = 2x = 20) johnsongrass gamete^6,8^. Tetraploid progenies do not produce vigorous rhizomes^8^ and do not over-winter well^9^, but some are fully fertile^8^ and can further hybridize with johnsongrass as a pollen or seed parent (i.e. backcrossing and introgression). Thus, opportunities for triploids to transfer their genes through backcrossing seem to be low compared to tetraploids, though this is yet to be tested. Interestingly, Hadley^8^ recovered one tetraploid and 25 triploid progeny when using emasculated diploid male fertile lines as the seed parent and suggested the possibility that cytoplasmic and genetic male sterile plants produce 2n gametes at a higher frequency.

Given that herbicide-tolerant (non-transgenic) lines are currently being developed in sorghum, there is a need to assess the upper limit of interspecific hybridization, the type of progeny produced and the effect of sorghum genotypes or sterility systems on these frequencies. This information is vital for developing gene flow mitigation strategies that limit the frequency of these events. As such, this greenhouse study was undertaken to i) determine the extent of interspecific hybridization occurring between male-sterile grain sorghum and johnsongrass and ii) classify the types of interspecific progeny from those crosses. Conceptually, the frequency of interspecific hybrids under controlled greenhouse conditions would represent the maximum level of outcrossing that could be expected between the two species.

## Materials and Methods

Two controlled crossing experiments, each done in a different environment (Experiment 1 in spring 2017 and Experiment 2 in summer 2017) were conducted using male-sterile sorghum genotypes that were pollinated with johnsongrass. The frequency of fertilized florets and of viable progeny were noted for each sorghum parent and the ploidy of all progeny was assessed.

### Plant germplasm

Two sorghum seed parent genotypes (Tx623 and Tx631) were selected as female parents. Tx623 is an inbred sorghum line developed and released in 1977 by Texas A&M Agrilife Research^10^. This line is used as a seed parent in commercial hybrids and is commonly used as a model in many genetic and genomic research studies^11^. Tx631 is an inbred tan plant with a white vitreous grain that was developed for the food industry^12^.

For each line, three different types of male sterility induction were assessed. First, cytoplasmic male sterile lines in A1 cytoplasm are available for both lines (designated as ATx623 and ATx631), hereafter referred to as CMS. Second, Tx623*ms*_3_ and Tx631*ms*_3_ are genetic male sterile sources wherein the genetic male sterile genotype is a homozygous recessive trait that is maintained in the population as a heterozygote (*Ms*_3_ *ms*_3_). In this case, male sterile (*ms*_3_ *ms*_3_) plants were identified at the start of flowering (designated as *ms*_3_ plants). Third, male sterility was induced by treating male fertile types with gametocide trifluoromethanesulfonamide (TFMSA) (Oakwood Chemical, Estill, SC). TFMSA was dissolved at a concentration of 20 mg/ml in an aqueous solution containing 5% glycerol and 0.25% tween 20^13^. A total of 1.5 ml of the TFMSA solution was applied to foliar tissue with a pipette 3 to 10 days prior to flag leaf emergence^14^. A locally occurring johnsongrass accession was used as the pollen parent in Experiment 1 which began in the spring of 2017. Experiment 2 began in the summer of 2017 the pollen parent being the F_5_ generation of an inbred line of johnsongrass with resistance to the acetyl coenzyme A carboxylaze (ACCase)-inhibitor herbicide fluazifop-p-ethyl (collected near West Memphis, AR). To increase the uniformity of the pollen source, all the johnsongrass plants were cloned from rhizomes.

### Growth conditions

Sorghum seeds were sown in 60 plastic pots (20 cm diameter) per genotype for experiment 1 over a six week period in February and March of 2017 and grown in a greenhouse. For Experiment 2, seed was sown in 30 plastic pots (20 cm diameter) per genotype in the month of June. Potting soil (Fafard 52) was used with 3 tablespoons of OSMOCOTE Pro 19-5-8 mixed in at the time of planting and an additional tablespoon added when the sixth leaf emerged. Five sorghum seeds were sown in each pot and plants thinned to one, two or three plants per pot depending on seedling emergence and development. The plants were irrigated as needed and insects were controlled using standard methods for greenhouse operations. Rhizomes of johnsongrass were planted in thirty 26 liter pots containing 6 tablespoons of OSMOCOTE Pro 19-5-8 added at the time of planting and 4 additional tablespoons 4 weeks later.

At flowering, johnsongrass, CMS and TFMSA induced male sterile sorghum plants were co-located in the same greenhouse while the ms_3_ sterile plants were moved to that same greenhouse upon confirmation of sterility. Prior to pollination, sorghum plants were examined at anthesis to verify male sterility; no male-fertile panicles were found among the TFMSA treated lines. All male-sterile panicles were thoroughly pollinated with johnsongrass pollen for at least two days with most plants receiving johnsongrass pollen over three days. Florets of each panicle were examined 20 to 25 days after pollination to assess total florets and florets with a developing seed. By 20 to 25 days after pollination a developing sorghum seed has expanded beyond the surrounding glumes and is easily seen. Mature seed from each panicle was harvested 35 to 40 days post pollination and planted individually in 72-cell trays. Ploidy was determined for each seedling by flow cytometry and confirmed with chromosome counts from a representative sample of each ploidy class.

Experiment 2 was conducted in the summer of 2017 in a different greenhouse using the same methodology but temperatures were distinctly warmer than they were in Experiment 1.

### Ploidy analysis

Leaf tissue (approx. 1 cm^2^) was harvested from young leaves of the progeny and chopped with a single-edged razor blade in cold, modified woody plant nuclei isolation buffer (WPB). WPB is an aqueous solution of 20 mM tris(hydroxymethyl)aminomethane (C_4_H_11_NO_3_), 4 mM magnesium chloride 6-hydrate (MgCl_2_ 6H_2_O), 2 mM ethylenedinitrilo-tetraacetic acid ([HO_2_CCH_2_]_2_ N[CH_2_]_2_N[CH_2_CO_2_-Na]_2_ H_2_O), 86 mM sodium chloride (NaCl), 10 mM sodium metabisulfite (Na_2_SO_3_), 1% polyvinylpyrrolidone (PVP-10) and 0.5% (v/v) Triton X-100 at pH 7.5^15^. RNase A (PureLink^TM^, Invitrogen, Carlsbad, CA) was added to WPB just prior to use at 5 mg L^−1^ to eliminate RNA. The slurry of each seedling sample was filtered through a 30 µm CellTrics disposable filter (Partec, Munster, Germany) and the nuclei in the filtered buffer was stained with 50 µg mL^−1^ propidium iodide (Sigma-Aldrich, St. Louis, MO). Samples were placed on ice until they were analyzed.

One sample of the propidium iodide labeled nuclei from leaf tissue of the 1043 seedlings in experiment 1 and 207 seedlings in experiment 2 were analyzed for ploidy on a BD Accuri C6 flow cytometer with an air-cooled laser operating at 488 nm. To confirm ploidy ratios of pentaploids and hexaploids two additional leaf samples were prepared and analyzed. Their fluorescence, collected through a 585/20 band pass filter (Becton Dickinson and Co., Franklin Lakes, NJ), was quantified and plotted along the x-axis of a histogram while counts of nuclei with the same value were plotted along the y-axis creating peaks. Since the fluorescence of each nucleus is directly proportional to its DNA quantity, the mean value of the peaks was used to determine ploidy ratios. All samples were compared to the nuclei of Tx623 with ploidy determined by the ratio of their respective G_1_ peaks.

**Figure 1:**
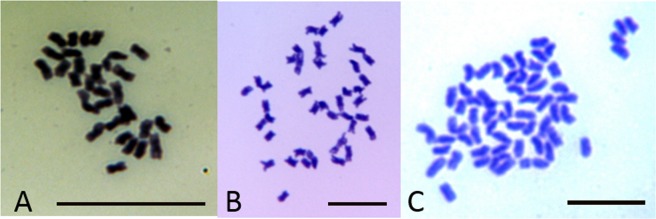
Metaphase chromosome spreads for progeny of sorghum x johnsongrass. (**A**) Triploid 2n = 3x = 30 chromosomes; (**B**) Tetraploid 2n = 4x = 40 chromosomes; (**C**) Hexaploid 2n = 6x = 60 chromosomes. Scale bars are 10 µm.

To confirm the ploidy levels assigned, mitotic chromosome spreads were produced using root tips from randomly selected seedlings of each ploidy type. Chromosome spreads of two diploid (2x) seedlings, two triploid (3x) seedlings, ten tetraploid (4x) seedlings, two pentaploid (5x) seedlings and two hexaploid (6x) seedlings were prepared using a modified protocol of Jewell and Islam-Faridi^16^ (Fig. 1). Actively growing root tips were excised from the plant and placed in an aqueous solution saturated with α-bromonaphthalene for 1.75 h in the dark at room temperature and then fixed overnight in a solution of 95% ethanol/glacial acetic acid (3:1 v/v). After fixation, the root tips were rinsed with deionized water several times over an hour, then hydrolyzed with 0.2 *M* HCl for 30 m at 37 °C in a water bath, followed by several rinses in deionized water. In preparation for maceration, cell walls were digested in an aqueous solution containing 30% v/v cellulase (C2730, Sigma-Aldrich, St. Louis, MO) and 15% pectinase (P2611, Sigma-Aldrich, St. Louis, MO). Root tips were placed on a clean glass slide in an ethanol/acetic acid (3/1) solution, macerated and spread with fine tipped forceps, air-dried overnight and stained with Azure Blue. Once dried, a glass cover slip was glued to the slide using Permount^®^. Chromosomes were viewed using a Zeiss Universal II microscope. Images of the chromosome spreads were obtained using a Microfire^TM^ digital camera (Optronics, www.optronics.com) and Pictureframe^TM^ software (Optronics). At least five metaphase cells were counted per seedling to classify ploidy.

### Statistical analysis

The data used in this study were checked for outliers using the Huber test in JMP 12.2.0 software (SAS Institute Inc., Cary, NC). To determine significance, an analysis of variance (ANOVA) was conducted using the following statistical model *Y* = *α*_*i*_ + *γ*_*i*_ + *δ*_*k*_ + *ε* where *Y* = triploid or tetraploid frequency, *α* = sorghum line (*i*), *γ* = week (*ι*), *δ* = sterility type ($\mathop{k}\limits_{\dot {}}$), and *ε* = error. After determining the significance of individual model effects, a comparison of means for lines and sterility was conducted in JMP using Tukey’s Honestly Significant Difference (HSD) test. Individual panicles were considered as replicates and progeny were analyzed by ploidy (triploid and tetraploid). The frequencies of the remaining ploidy categories were simply too small for effective statistical discrimination. Likewise, in the summer environment, blocking by panicle was not completed due to low relative seed set because of excessive heat (38 °C day, 28 °C night and 14 hour photoperiod). Thus, data from the second environment is used for relative comparison to the first environment.

## Results and Discussion

### Rates of interspecific hybridization

In Experiment 1, a total of 199,820 florets on 335 panicles were pollinated with johnsongrass pollen**;** 176,295 of these florets had a developing seed (Table 1). The number of florets with a developing seed was similar for both sorghum genotypes (Tx623 and Tx631), averaging 88%. The presence of the developing seed indicates fertilization occurred and an embryo was developing, therefore a pre-fertilization barrier was not present in these crosses. At maturity, a total of 1,276 fully developed seeds were harvested. The relatively low frequency of mature seed compared to developing seed implies the presence of a post-fertilization barrier. In fact, most (175,019/176,295) of the developing seeds began to collapse between 18 and 21 days after pollination and were composed primarily of a seed coat at maturity. While it was not possible to confirm the ploidy of these collapsed hybrids, they were most likely triploid and succumbed to the triploid block wherein the endosperm begins to degenerate sometime in the second week of development. The embryo, which obtains its nutrients from the endosperm, ceases developing and eventually dies^17,18^.

**Table 1:** Seed development characteristics from male sterile panicles and florets of two *S. bicolor* genotypes pollinated with johnsongrass (*S. halepense*) pollen in a greenhouse in College Station, Texas in the Spring of 2017. Male sterility was induced by either cytoplasmic male sterility (CMS), genetic male sterility (*ms*_3_) or chemical gametocide (TFMSA).

Genotype/Sterility Type	No. panicles with		Total Florets	Seed		Ploidy of viable progeny						
	Seed	No Seed		Developing	Mature	2x	3x		4x		5x	6x
ATx623(CMS)	59	10	39,607	35,103	467	15	8	**a**^**a**^	370	**a**^**a**^	2	3
BTx623*ms*_3_	30	17	29,433	26,812	91	19	0	**a**	44	**b**	0	2
BTx623(TFMSA)	50	6	32,913	28,891	598	1	1	**a**	483	**a**	3	2
Sum	139	33	101,953	90,806	1156	35	9	**A**^**b**^	897	**A**^**b**^	5	7
ATx631(CMS)	25	55	45,777	39,763	58	12	4	**a**	25	**b**	0	0
BTx631*ms*_3_	11	17	11,443	10,053	18	6	5	**a**	6	**b**	1	0
BTx631(TFMSA)	24	31	40,647	35,673	43	7	5	**a**	18	**b**	1	0
Sum	60	103	97,867	85,489	120	25	14	**A**	49	**B**	2	0
Totals	199	136	199,820	176,295	1276	60	23		946		7	7

^a^Different lowercase letters within a genotype indicate statistical differences between the frequency of progeny based on male sterility type.

^b^Different uppercase letters between genotypes indicate statistical differences between the frequency of derived progeny combined across male-sterility types.

Of the 1,276 harvested seed, 1,043 germinated and grew sufficiently for ploidy analysis. By frequency of seedlings, 5% were diploid, 2% were triploid, 91% were tetraploid and 1% each were pentaploid and hexaploid. While the number of florets with a developing seed was similar, the number of fully developed seed harvested from the two seed parents differed significantly, 1156 seed from Tx623 and 120 seed from Tx631. Tx623 produced significantly more tetraploid progeny than Tx631 (Table 1); no other differences in ploidy frequency were detected between the two lines. Within the seed parents, differences in frequency of hybrids were detected by sterility type in Tx623, with *ms*_3_ male sterile plants producing significantly lower frequencies of tetraploid progeny than either the CMS or TFMSA-induced sterile lines. No other differences were detected within Tx623 lines nor within Tx631 lines.

Of all crosses involving Tx623, 0.99% of fertilized florets were tetraploid compared to 0.06% of Tx631. At least one tetraploid seedling was recovered from 136 of the 172 panicles of Tx623 that had been pollinated with johnsongrass. The maximum number of tetraploids recovered from a single panicle was 58, representing 4.6% of the developing seed on that panicle. In comparison, tetraploids were recovered from only 32 of the 163 panicles of Tx631 that were pollinated with johnsongrass, the most from a single panicle being 4 which is 0.6% of the developing seed for that panicle.

In Experiment 2, the relative numbers of florets pollinated (61,478) and the progeny produced (207) were lower than in Experiment 1, and could be attributed to the higher summer temperatures of the second environment (Table 2). However, the trends were comparable for most ploidy levels except for an increase in diploid progeny (Table 2). All diploid seedlings were sprayed with fluazifop-p-ethyl herbicide to identify hybrids. It is expected that hybrids would have acquired resistance to the herbicide from the johnsongrass parent. None of the diploids survived the herbicide application suggesting that there were no hybrids among the diploid progeny. Consequently, these diploids were likely the result of fertility reversion in Tx631 mediated by high temperatures. Temperature-mediated fertility reversion has been reported previously in sorghum^19^.

**Table 2:** Seed development characteristics from male sterile panicles and florets of two *S. bicolor* genotypes pollinated with johnsongrass (*S. halepense*) pollen in a greenhouse in College Station, Texas in the Summer of 2017. Male sterility was induced by either cytoplasmic male sterility (CMS), genetic male sterility (*ms*_3_) or chemical gametocide (TFMSA).

Genotype/Sterility Type	No. panicles with		Total Florets	Seed		Ploidy of viable progeny				
	Seed	No Seed		Developing	Mature	2x	3x	4x	5x	6x
ATx623(CMS)	12	12	7,056	3,081	30	0	2	15	0	0
BTx623*ms*_3_	0	5	1,038	602	0	0	0	0	0	0
BTx623(TFMSA)	20	14	7072	2803	132	0	0	47	0	0
Sum	32	31	15,166	6,486	162	0	2	62	0	0
ATx631(CMS)	7	31	13,130	5,148	8	2	1	0	0	0
BTx631*ms*_3_	8	33	13,133	5,127	59	41	0	3	0	1
BTx631(TFMSA)	8	38	20,049	7,522	106	93	1	1	0	0
Sum	23	102	46,312	17,797	173	136	2	4	0	1
Totals	62	155	61,478	24,283	335	136	4	66	0	1

### Effect of sterility on interspecific hybridization

Tetraploid production was not influenced by the type of sterility induction. Whether sterility was from CMS, GMS or induced by TFMSA, no differences in the tetraploid frequency was detected among sterility types within Tx631. However, the tetraploid frequency in BTx623*ms*_3_ was lower than the other two sterility induction systems. It was somewhat surprising that BTx623*ms*_3_ markedly differed in the number of recovered tetraploids when compared to that of the A- and B- lines, however, this observation was the same for Experiment 2 (Table 2). Since the *ms*_3_ genotypes were grown in a different greenhouse in spring 2017 until sterility was confirmed, it could be speculated that there was an environmental effect, but results of Experiment 2 were similar for the production of tetraploid progeny where all genotypes were grown in the same greenhouse.

TFMSA applied to the B-lines of Tx623 and Tx631 functioned well as a male sterility inducer in Experiment 1 as fewer diploids were recovered from these lines than from the male sterile lines. However, in Experiment 2 most of the seedlings recovered from TFMSA treated BTx631 and BTx631*ms*_3_ were diploid. This is the first time B-lines could be used to effectively test for hybridization with johnsongrass as previous studies were limited by time-consuming mechanical emasculations or difficult hot water treatments. When comparing CMS and TFMSA induced male sterile lines within genotypes, there was no difference in the frequency of 2n gametes produced.

### Ploidy groups and their derivation

Most hybrids of sorghum х johnsongrass are expected to be triploid as normal haploid gametes for sorghum and johnsongrass would have n = x = 10 and n = 2x = 20 chromosomes, respectively, producing 2n = 3x = 30 progeny. The triploid block likely explains the reduced frequency of triploid embryos that fully develop into viable seed. Tetraploid hybrids of sorghum x johnsongrass likely result from the union of a 2n gamete of sorghum and a normal gamete of johnsongrass. Unreduced gametes can result from either pre- or post-meiotic chromosome doubling during mitosis, but have more commonly been a result of a restitution of the nuclear DNA during the first or second division of meiosis^20^. Because these hybrids are tetraploids, it is likely they are fertile and fully sexually compatible with johnsongrass.

The other ploidy types observed among the progeny occurred at much lower frequencies. The largest class was diploid and we surmise that most of these progeny were the result of either self-pollination or cross pollination with *S. bicolor* as was confirmed in the previously described evaluation for herbicide tolerance. While the panicles were visually male sterile, it is possible that individual florets reverted to fertility. These revertants are known to occur in CMS and TFMSA sterility induction employed in this study^14,21,22^. The hexaploid progeny recovered in this study could result from a chromosome doubling of a triploid hybrid or the union of 2n gametes (2n = 2x = 20 and 2n = 4x = 40). Numerous examples of polyploidy arising from 2n gametes have been reported, but very few reports exist on somatic chromosome doubling^1^. Interestingly, the frequency of hexaploids was similar to triploids, and may imply that doubling of a triploid might stabilize the embryo and its development. Hadley^8^ also reported the presence of hexaploids and even higher chromosome numbers in interspecific hybrids of one of the crosses. Finally, pentaploid hybrids (2n = 5x = 50) did occur, but there is no obvious cytological path to their presence. Chromosome doubling in the embryo is not a valid explanation as that would produce a progeny with an even ploidy, but it could result from a 2n gamete of johnsongrass uniting with a 1n gamete of sorghum. However, this study was not designed to account for this occurrence so further evaluation will be required to confirm the exact cause of the pentaploid progeny.

### Genotypic effect on interspecific hybridization

There was a clear difference between Tx623 and Tx631 in the frequency of tetraploid progeny recovered in both crossing environments, but no differences for triploid, pentaploid or hexaploid progeny. Given that tetraploid progeny are most likely derived from the union of a 2n gamete from sorghum and a haploid gamete from johnsongrass, the results indicate that 2n gamete formation regularly occurs in sorghum ovules and that the frequency of production varies with genotype. This is consistent with previous observations in other species; 2n gamete production is genetically controlled and expression is affected by both genetic and environmental effects^23–25^. In this study, if we assume that every 2n gamete produced a tetraploid progeny, the frequency of 2n gametes averaged ~1% of florets per panicle and varied from 0 to 6% among individual Tx623 panicles and 0 to 0.7% in Tx631 panicles.

This is the first study to assess the frequency of 2n gametes in sorghum and the frequencies reported herein appear to be relatively high given the limited reports of their presence in sorghum. Previous reports agree that sorghum x johnsongrass progeny are far more likely to be tetraploid than triploid (a total of 27 triploids and 164 tetraploids were recovered)^6,8,9,17,26^. This implies the presence of 2n gametes, but the numbers of florets pollinated and progeny obtained in those studies were too small to make any specific inferences about 2n gamete frequency. There is one notable exception; Hadley^8^ recovered 24 triploids and 2 tetraploid progeny (sorghum x johnsongrass) when hot water was used to induce male sterility. It is not known if the treatment could have caused these differential results.

There is a question as to why 2n gametes have not been noted in sorghum given their apparent commonality; the likely explanation is that a tetraploid pollinator is essential to effectively identify them. For example, in a normal sorghum pollination, the vast majority of sorghum florets are either self-pollinated or cross-pollinated with *S. bicolor* pollen. In this case, a 2n female gamete would be fertilized with a normal haploid gamete from *S. bicolor* to produce a triploid embryo. As noted previously, triploid sorghum embryos usually abort due to the triploid block. A tetraploid seedling would be extremely rare and not recognized as tetraploid or would be considered an off type and undesirable if phenotypically distinct. Thus it would most likely be eliminated during the breeding process. As such, no evidence of the 2n gamete remains. In this study the sorghum panicle received only *S. halepense* pollen, which served as an effective screen for identifying 2n gametes in these genotypes.

Unreduced gametes in sorghum seed parents have specific consequences with regard to the production of interspecific sorghum x johnsongrass hybrids. Specifically, tetraploid progeny from sorghum x johnsongrass are likely to be both female and male fertile^8^ which would facilitate gene flow into feral or weedy relatives. Triploids however, are typically male sterile, but may be female fertile and thus can still facilitate gene flow via hybridization to some extent. Given that differences exist between sorghum lines for 2n gamete formation, it appears that specific selection of sorghum seed parents for low rates of 2n gamete formation would reduce the likelihood of tetraploid hybrid formation, and thereby the risk and adverse consequences of gene flow.

This study specifically eliminated *S. bicolor* pollen competition for two reasons. First, testing without pollen competition establishes the maximum rate of interspecific hybridization (~1% noted in this study) that would be expected to occur in sorghum х johnsongrass. In the presence of *S. bicolor* pollen, the frequency of interspecific hybrids will naturally decline. Further studies are needed to fully understand the level of reduction that can be expected.

The second reason that *S. bicolor* pollen competition was not included is because the male-sterile sorghum x johnsongrass hybridization may be an important method of gene flow between sorghum and johnsongrass. When hybrid grain sorghum is harvested and transported to markets some of the grain is lost in the production field or during transport, serving as a founding seed for the establishment of feral sorghum plants. Given that these plants were derived from hybrids produced with cytoplasmic male sterility, approximately 25% of them will be male sterile and thus would present the exact scenario of a male sterile *S. bicolor* seed parent being primarily pollinated by *S. halepense*. Feral sorghum plants are found co-existing with johnsongrass in established transportation routes^27^. This scenario also occurs in production fields when volunteer sorghum is established from seed lost during harvest.

The dynamics of gene flow must be fully understood in sorghum in order to avoid what has occurred in the US with Clearfield® rice (*Oryza sativa* L.) where gene flow from herbicide-resistant cultivated rice to weedy rice greatly diminished the long-term effectiveness of this technology. The Clearfield® system with resistance to imidazolinone herbicides was introduced in the US in 2002 to selectively control grass weeds, particularly weedy rice, in rice production^28,29^. This traditionally bred trait was rapidly accepted by growers, but within five years there were growing signs of outcrossing and gene escape to weedy red rice^29^. To avoid this scenario in sorghum, a thorough understanding of crop/weed hybridization as well as mitigation strategies are critical^30^.

## Conclusions

The results herein indicate that the frequency of viable seed from an interspecific cross of *S. bicolor* x *S. halepense* is variable and in this study ranged from 0.1 to 1%. Given that these studies were conducted in controlled and optimum conditions and without *S. bicolor* pollen present, the actual outcrossing rate in natural conditions is expected to be much lower. However, if interspecific hybrids are produced, they are most likely to produce fertile tetraploid progeny which increases the opportunities for gene flow to occur between the two species. Further, differences in 2n gamete formation among Tx623 and Tx631 imply that the use of seed parents with lower rates of 2n gamete formation may reduce the likelihood of gene flow. Additional studies are essential to assess not only the rates of interspecific hybridization under field conditions, but also the fitness and fertility of the progeny produced from these hybridization events.

## Supplementary information

Dataset 1

Dataset 2

## Data Availability

All data generated or analyzed during this study are included in this published article (and its Supplementary Information Files).
